# Effects of subanesthetic intravenous ketamine infusion on neuroplasticity-related proteins in the prefrontal cortex, amygdala, and hippocampus of Sprague-Dawley rats

**DOI:** 10.1016/j.ibror.2019.01.006

**Published:** 2019-01-16

**Authors:** Michael Zhang, Kennett D. Radford, Mercedes Driscoll, Salsabila Purnomo, Jean Kim, Kwang H. Choi

**Affiliations:** a Department of Psychiatry, Uniformed Services University, 4301 Jones Bridge Road, Bethesda, MD 20814, United States; b Center for the Study of Traumatic Stress, Uniformed Services University, 4301 Jones Bridge Road, Bethesda, MD 20814, United States; c Daniel K. Inouye Graduate School of Nursing, Uniformed Services University, 4301 Jones Bridge Road, Bethesda, MD 20814, United States; d National Capital Consortium Psychiatry Residency Program, Walter Reed National Military Medical Center, Bethesda, MD 20814, United States

**Keywords:** Intravenous ketamine, Fear conditioning, Brain-derived neurotrophic factor, c-Fos, Extracellular signal-regulated kinase, Prefrontal cortex

## Abstract

Ketamine, a multimodal dissociative anesthetic, is a powerful analgesic administered following trauma due to its hemodynamic and respiratory stability. However, ketamine can cause hallucination and dissociation which may adversely impact traumatic memory after an injury. The effects of ketamine on proteins implicated in neural plasticity are unclear due to different doses, routes, and timing of drug administration in previous studies. Here, we investigated the effects of a single intravenous (IV) ketamine infusion on protein levels in three brain regions of rats. Adult male Sprague-Dawley rats with indwelling IV catheters underwent an auditory fear conditioning (three pairings of tone and mild footshock 0.8 mA, 0.5 s) and received a high dose of IV ketamine (0 or 40 mg/kg/2 h) infusion (Experiment 1). In a follow-up study, animals received a low dose of IV ketamine (0 or 10 mg/kg/2 h) infusion (Experiment 2). Two hours after the infusion, brain tissue from the medial prefrontal cortex (mPFC), hippocampus, and amygdala were collected for western blot analyses. Protein levels of a transcription factor (c-Fos), brain-derived neurotrophic factor (BDNF), and phosphorylated extracellular signal-regulated kinase (pERK) were quantified in these regions. The 40 mg/kg ketamine infusion increased c-Fos levels in the mPFC and amygdala as well as pERK levels in the mPFC and hippocampus. The 10 mg/kg ketamine infusion increased BDNF levels in the amygdala, but decreased pERK levels in the mPFC and hippocampus. These findings suggest that a clinically relevant route of ketamine administration produces dose-dependent and brain region-specific effects on proteins involved in neuroplasticity.

## 1 Introduction

Health care providers clinically administer ketamine, a non-competitive *N*-methyl-D-aspartate (NMDA) glutamate receptor antagonist, to provide analgesia and anesthesia (Harrison and Simmonds, 1985). Ketamine has recently exhibited an upswing in popularity due to its ability to act as an effective trauma analgesic in the pre-hospital setting. Its cardiovascular and respiratory stability along with its high safety ceiling makes ketamine an attractive analgesic to administer following a traumatic injury (Morris et al., 2009; Radvansky et al., 2015). However, psychomimetic properties of ketamine may potentiate post-traumatic stress disorder (PTSD) symptoms for survivors of physical trauma who received ketamine treatment (Honsberger et al., 2015). Preclinical and clinical investigations have yielded inconsistent results on the effects of ketamine on PTSD. For instance, clinical investigations have reported that peri-trauma ketamine administration decreased (McGhee et al., 2014), increased (Winter and Irle, 2004; Schonenberg et al., 2005), or had no effect (McGhee et al., 2008; Mion et al., 2017) on PTSD or acute stress disorder (ASD) symptoms. Meanwhile, preclinical studies have shown increased fear behaviors when ketamine was administered after the stress exposure or fear learning in rodents (Juven-Wetzler et al., 2014; Saur et al., 2017; Radford et al., 2018a). These data suggest that ketamine administration following trauma may exacerbate fear and stress behaviors.

Most preclinical studies utilize an intraperitoneal (IP) ketamine injection to rodents, which may not translate well to clinical studies of intravenous (IV) ketamine administration. An IV ketamine infusion can be beneficial because it maintains a steady-state drug plasma concentration over an extended time period for analgesia. In contrast, a bolus ketamine IP injection in rodents results in a shorter duration of action due to its short half-life (Palenicek et al., 2011). We found that the route of ketamine administration (IV vs. IP) induced opposite effects on fear behaviors in rats (Radford et al., 2018a). Therefore, it is important to note the differences between the route of ketamine administration on brain function and stress related behaviors.

We reported that an IV ketamine infusion dose-dependently increased stress hormone corticosterone (CORT) levels and decreased brain-derived neurotrophic factor (BDNF) levels in the plasma samples of rats (Radford et al., 2018b). Two hours after IV ketamine infusion, the CORT levels returned to a baseline, while the BDNF levels were decreased. This suggests that IV ketamine infusion activates the hypothalamic-pituitary-adrenal (HPA) axis and downstream stress pathways. Therefore, it is important to investigate IV ketamine infusion effects on brain protein levels that are implicated in stress and plasticity.

Ketamine is known to impact multiple signaling pathways and proteins implicated in synaptic plasticity and memory formation. The transcription factor c-Fos, a general marker for neuronal activity, has been implicated in fear and stress. Administration of NMDA receptor antagonists including ketamine has shown to increase c-Fos protein expression in the brain regions that are involved in the regulation of memory (Nakao et al., 2002; Pietersen et al., 2006; Peng et al., 2011; Nowak et al., 2012). In addition, the BDNF has an important role as it acts on certain neurons of the brain to support neuronal survival, differentiation, and synapse formation (Akinfiresoye and Tizabi, 2013). Recent studies have attributed lower levels of BDNF to be a key factor in the development of PTSD (Dell’Osso et al., 2009; Palenicek et al., 2011; Su et al., 2015). However, preclinical studies on BDNF are inconsistent as they reported either increased (Garcia et al., 2008b; Ibla et al., 2009), decreased (Fraga et al., 2013; Ke et al., 2014) or had no change (Garcia et al., 2008a; Saur et al., 2017) after ketamine administration. Moreover, phosphorylation of extracellular signal-regulated kinase (pERK) is critical for the development of long-term memory (Adams and Sweatt, 2002). Preclinical studies have reported that fear conditioning (Atkins et al., 1998) and IP ketamine (10 and 30 mg/kg) injection (Li et al., 2010; Fernandes and Li, 2017) increased pERK levels in rodent brains. However, the effects of IV ketamine infusion on pERK levels in the brain are not known.

The purpose of this investigation was to determine the effects of subanesthetic doses of an IV ketamine (10 and 40 mg/kg/2 h) infusion on the levels of c-Fos, BDNF, and pERK in brain regions that are critical for neuroplasticity. We hypothesized that low and high doses of ketamine may differentially regulate c-Fos, BDNF, and pERK levels in the mPFC, amygdala, and hippocampus of rats. To our knowledge, this is the first study to report the effects of IV ketamine infusion on plasticity related protein levels in these brain regions of rats.

## 2 Methods

### 2.1 Animals

Adult male Sprague-Dawley rats weighing 300–325 g at the time of arrival were used for this study (Envigo; Dublin, VA). A total of 64 rats were individually housed in a rectangular Plexiglas cage in a climate-controlled room with a 12-h reverse dark-light cycle (lights on at 6 p.m. and off at 6 a.m.). Throughout the experiments, standard drinking water and rat chow were available ad libitum. Animals were acclimated and handled daily for seven days before the experiments. All experiments were conducted during the dark cycle. Animal use and procedures were in accordance with National Institutes of Health *Guide for the Care and Use of Laboratory Animals* and Use Committee at the Uniformed Services University of the Health Sciences (Bethesda, MD).

### 2.2 Intravenous catheter surgery

A jugular venous catheter (3Fr, polyurethane; Instech, Plymouth Meeting, PA) was surgically placed under isoflurane anesthesia by personnel at Envigo Laboratories prior to arrival. The catheter was tunneled under the skin and connected to a vascular access button (Instech, Plymouth Meeting, PA) that exited the dorsal position between the front rodent scapulae. The catheter was flushed once every three days with a 0.1 mL heparin/glycerol solution (Braintree Scientific; Braintree, MA) to maintain venous patency.

### 2.3 Experimental design

Experiment 1: Animals were randomly assigned to four groups (n = 10–12 per group): No fear conditioning & IV saline (Group 1), no fear conditioning & IV ketamine (Group 2), fear conditioning & IV saline (Group 3), and fear conditioning & IV ketamine (Group 4). Ketamine hydrochloride (100 mg/mL) was obtained from Mylan Institutional LLC and was diluted to 5 mg/mL in 0.9% saline. Group 2 and Group 4 received a 5 mg/kg IV ketamine bolus and a 2-h ketamine (40 mg/kg) infusion. Group 1 and Group 3 received a saline bolus and a 2-h saline infusion (1 mL/h).

Experiment 2: Animals were randomly assigned to two groups (n = 8 per group): IV saline (Group 1) and IV ketamine (Group 2). Group 1 received a saline bolus and a 2-h saline infusion (1 mL/h). Group 2 received a 2 mg/kg IV ketamine bolus and a 2-h ketamine (10 mg/kg) infusion. All ketamine and saline bolus doses were delivered in a 1 mL/kg volume.

### 2.4 Auditory fear conditioning

The auditory fear conditioning chamber was constructed with aluminum walls and Plexiglas, equipped with a house light (2–3 lx) and a mounted camera to allow recording during testing (Coulbourn Instruments, Lehigh Valley, PA). The chamber contained a metal rod floor connected to a shock generator (Coulbourn Instruments, Lehigh Valley, PA) and a speaker mounted in the wall to provide auditory stimuli (Coulbourn Instruments, Lehigh Valley, PA). The chamber was housed in a larger sound-attenuating box with a background noise level of 55 dB. After a 180-s acclimation period, rats received three pairings of an auditory tone (5 kHz, 75 dB, 20 s) that co-terminated with a mild electric footshock (0.8 mA, 0.5 s). An inter-trial interval (ITI) ranging from 90 to 120 s prevented tone prediction in animals. Freezing behavior was scored during the 20-s tone delivery to ensure rats acquired adequate fear learning. One minute after the final tone and shock pairing, animals were then removed from the chamber. Animals with no fear conditioning (Group 1 and Group 3) received the same auditory tone without the footshock during the session.

### 2.5 IV ketamine infusion

Racemic (±) ketamine hydrochloride (100 mg/mL) (Mylan Institutional LLC, Rockford, IL) was diluted in 0.9% sterile saline and was administered to an individual rat placed in an infusion chamber (Med Associates Inc., St. Albans, VT). Each chamber was equipped with an infusion pump (Harvard Pump 11 Elite, Holliston, MA) using a 5 mL Hamilton glass syringe connected to a fluid swivel (Instech, Plymouth Meeting, PA) by a polyurethane tubing encased in a metal spring-wire tether (Instech, Plymouth Meeting, PA). The spring wire tether was attached to the vascular access button on the rat using a luer-lock connection. Each tethered rat had free mobility within the chamber during the 2-h infusion period. A dim red light illuminated each box to allow observation of rat’s behavior.

### 2.6 Western blot analysis

Brain tissue was collected 2 h after the infusion. The medial prefrontal cortex (mPFC), amygdala, and dorsal hippocampus based on the rat brain atlas (Paxinos and Watson, 1998) were rapidly dissected using a brain matrix on wet ice. All samples were immediately frozen on dry ice and stored at −80 °C. Samples were homogenized in RIPA buffer containing 0.22% Beta glycerophosphate, 10% Tergitol-NP40, 0.18% Sodium orthovanadate, 5% Sodium deoxycholate, 0.38% EGTA, 1% SDS, 6.1% Tris, 0.29% EDTA, 8.8% Sodium chloride, and 1.12% Sodium pyrophosphate decahydrate. The protein concentration was quantified using the BCA assay (Thermo Fisher Scientific, Waltham, MA) and an equal amount of protein samples were loaded and separated on an SDS-Page gel (NuPage 4–12% Bis-Tris gel, Thermo Fisher Scientific, Waltham, MA). Following electrophoresis, proteins were transferred to a nitrocellulose membrane and blocked for 1 h in 5% milk TBS-T (TBS and 0.1% Tween 20). After the blocking, membranes were incubated with a primary antibody at 4 °C overnight. The following primary antibodies were used: BDNF (1:2,000 Santa Cruz Biotechnology, Dallas, TX), c-Fos (1:1000 Biolegend, San Diego, CA), pERK (1:2000 Biolegend, San Diego, CA), and beta-actin (1:200,000 Abcam, Cambridge, United Kingdom). Next, the membranes were washed in TBS-T for 30 min (10 min × 3 times) and incubated with HRP-conjugated secondary antibody for 1 h. The protein bands were detected using the chemiluminescence method (Bio-Rad, Hercules, CA) with ChemiDoc equipment (Bio-Rad, Hercules, CA). After the target protein quantification, the membranes were stripped for 15 min using the Restore™ Western Blot Stripping Buffer (Thermo Fisher Scientific, Waltham, MA). The stripped membranes were used to probe for a reference protein (beta-actin) to adjust for the variations in protein amount and sample loading.

### 2.7 Statistics

The intensities of the protein bands were quantified by using the Image-Lab software (Bio-Rad, Hercules, CA). All data are presented as mean ± standard error of the mean (SEM) and were analyzed using GraphPad Prism (GraphPad Software Version 7.0). For Experiment 1, a two-way analysis of variance (ANOVA) with fear conditioning and ketamine as independent variables and Tukey’s post-hoc tests were used to compare group differences. For Experiment 2, independent samples *t*-test was used to compare saline and ketamine groups. The accepted level of significance was p < 0.05.

## 3 Results

**Fig. 1:**
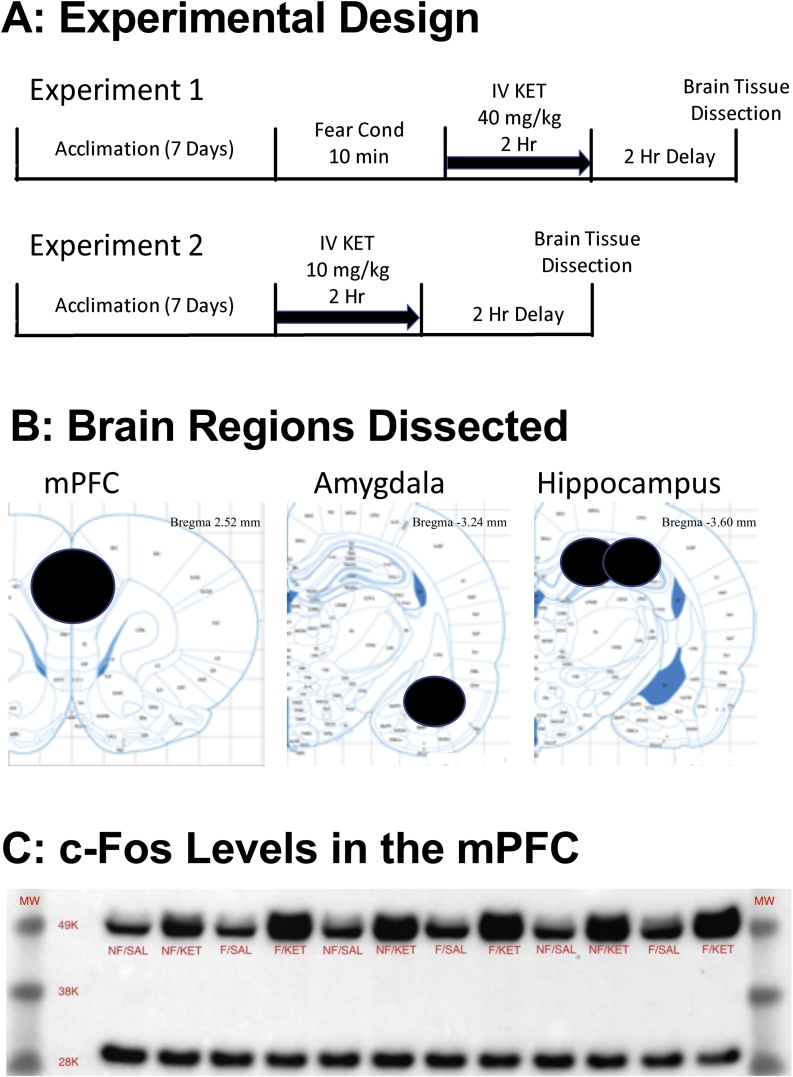
A: The study design for Experiment 1 and Experiment 2. B: Brain regions dissected for western blot analysis. C: An example image of western blot showing increased c-Fos levels 2 h after a high dose of ketamine infusion (40 mg/kg/2 h). NF/SAL: No fear conditioning & IV saline infusion, NF/KET: No fear conditioning & IV ketamine infusion, F/SAL: Fear conditioning & IV saline infusion, F/KET: Fear conditioning & IV ketamine infusion.

The Fig. 1A illustrates the experimental design of the study. The three brain regions dissected and analyzed in the current study are depicted in Fig. 1B. The Fig. 1C shows an example western blot image of c-Fos in the mPFC. Regardless of the fear conditioning, 40 mg/kg ketamine infusion groups (Groups 2 and 4) showed increased c-Fos levels in the mPFC as compared to the saline infusion groups (Groups 1 and 3).

**Fig. 2:**
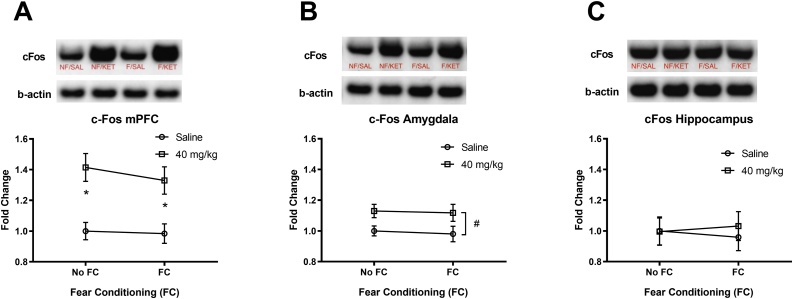
The effects of a high dose IV ketamine (40 mg/kg/2 h) on c-Fos protein levels in the mPFC, amygdala, and hippocampus of rats. A: IV ketamine increased c-Fos levels in the mPFC. B: IV ketamine increased c-Fos levels in the amygdala, C: IV ketamine did not alter c-Fos levels in the hippocampus. NF/SAL: No fear conditioning & IV saline infusion, NF/KET: No fear conditioning & IV ketamine infusion, F/SAL: Fear conditioning & IV saline infusion, F/KET: Fear conditioning & IV ketamine infusion. Data shown as mean ± SEM (*p < 0.05). A two-way ANOVA main effect of ketamine (#p < 0.05).

The effects of fear conditioning and a high dose ketamine (40 mg/kg) infusion on c-Fos levels in brain regions are shown in Fig. 2. A two-way ANOVA for c-Fos in the mPFC showed no significant interaction (fear conditioning × ketamine) and no main effect of fear conditioning, but a significant main effect of ketamine F(1, 32) = 24.71, p < 0.0001 (Fig. 2A). Tukey’s post-hoc tests revealed significant differences between No FC/SAL and No FC/KET (adj. p = 0.003), and FC/SAL and FC/KET (adj. p = 0.015). A two-way ANOVA for c-Fos in the amygdala showed no significant interaction (fear conditioning × ketamine) and no main effect of fear conditioning, but a significant main effect of ketamine F(1, 32) = 8.33, p = 0.007 (Fig. 2B). However, Tukey’s post-hoc tests revealed no significant differences between the groups (p > 0.05). A two-way ANOVA for c-Fos in the hippocampus showed no significant interaction (fear conditioning × ketamine) and no main effect of fear conditioning and ketamine (Fig. 2C). Overall, a high dose ketamine (40 mg/kg) infusion increased c-Fos levels in the mPFC and amygdala compared to the saline controls.

**Fig. 3:**
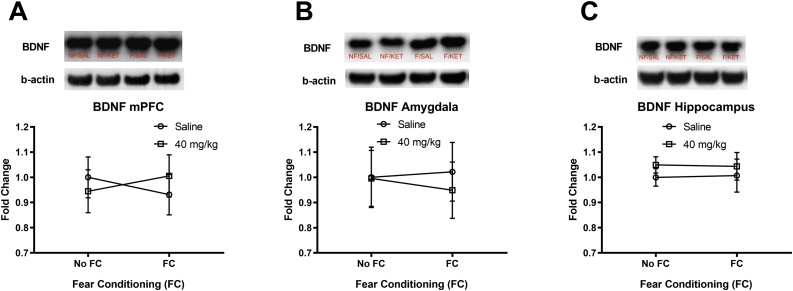
The effects of a high dose IV ketamine (40 mg/kg/2 h) on BDNF protein levels in the mPFC, amygdala, and hippocampus of rats. A: IV ketamine had no effect on BDNF levels in the mPFC. B: IV ketamine had no effect on BDNF levels in the amygdala, C: IV ketamine had no effect on BDNF levels in the hippocampus. NF/SAL: No fear conditioning & IV saline infusion, NF/KET: No fear conditioning & IV ketamine infusion, F/SAL: Fear conditioning & IV saline infusion, F/KET: Fear conditioning & IV ketamine infusion. Data shown as mean ± SEM.

The effects of fear conditioning and a high dose ketamine (40 mg/kg) infusion on BDNF levels in brain regions are shown in Fig. 3. A two-way ANOVA for BDNF in the mPFC showed no significant interaction (fear conditioning x ketamine) and no main effect of fear conditioning and ketamine (Fig. 3A). A two-way ANOVA for BDNF in the amygdala showed no significant interaction (fear conditioning × ketamine) and no main effect of fear conditioning and ketamine (Fig. 3B). A two-way ANOVA for BDNF in the hippocampus showed no significant interaction (fear conditioning × ketamine) and no main effect of fear conditioning and ketamine (Fig. 3C). Overall, a high dose ketamine (40 mg/kg) infusion did not alter BDNF levels in the mPFC, amygdala, or hippocampus compared to the saline controls.

**Fig. 4:**
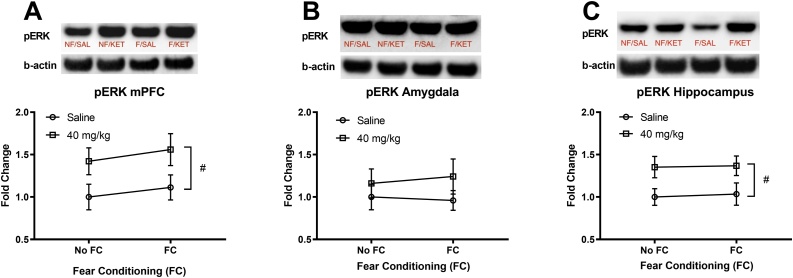
The effects of a high dose IV ketamine (40 mg/kg/2 h) on pERK protein levels in the mPFC, amygdala, and hippocampus of rats. A: IV ketamine increased pERK levels in the mPFC. B: IV ketamine had no effect on pERK levels in the amygdala, C: IV ketamine increased pERK levels in dorsal hippocampus. NF/SAL: No fear conditioning & IV saline infusion, NF/KET: No fear conditioning & IV ketamine infusion, F/SAL: Fear conditioning & IV saline infusion, F/KET: Fear conditioning & IV ketamine infusion. Data shown as mean ± SEM. A two-way ANOVA main effect of ketamine (#p < 0.05).

The effects of fear conditioning and a high dose ketamine (40 mg/kg) infusion on pERK levels in brain regions are shown in Fig. 4. A two-way ANOVA for pERK in the mPFC showed no significant interaction (fear conditioning × ketamine) and no main effect of fear conditioning, but a significant main effect of ketamine F(1, 32) = 7.186, p = 0.012 (Fig. 4A). However, Tukey’s post-hoc tests revealed no significant differences between the groups (p > 0.05). A two-way ANOVA for pERK in the amygdala showed no significant interaction (fear conditioning × ketamine) and no main effect of fear conditioning and ketamine (Fig. 4B). A two-way ANOVA for pERK in the hippocampus showed no significant interaction (fear conditioning × ketamine) and no main effect of fear conditioning, but a significant main effect of ketamine F(1, 32) = 8.428, p = 0.007 (Fig. 4C). However, Tukey’s post-hoc tests revealed no significant differences between the groups (p > 0.05). Overall, a high dose ketamine (40 mg/kg) increased pERK levels in the mPFC and hippocampus compared to the saline controls.

**Fig. 5:**
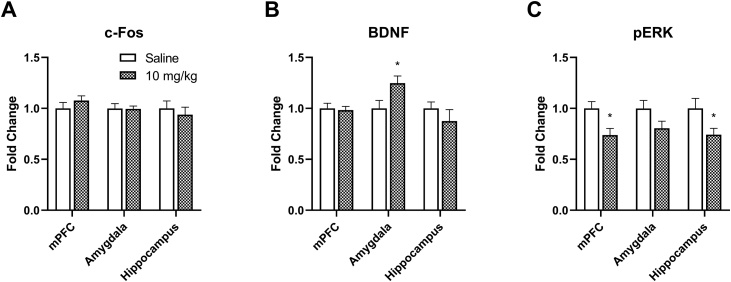
The effects of a low dose IV ketamine infusion (10 mg/kg/2 h) on c-Fos, BDNF, and pERK levels in the mPFC, amygdala, and hippocampus of rats. A: The IV ketamine infusion did not alter c-Fos levels in the mPFC, amygdala, and hippocampus. B: The IV ketamine infusion selectively increased BDNF levels in the amygdala, but not in mPFC and hippocampus of rats. C: The IV ketamine infusion reduced pERK levels in the mPFC and hippocampus, but not in amygdala of rats. Data shown as mean ± standard error of mean. (*p < 0.05).

The effects of a low dose ketamine (10 mg/kg) infusion on c-Fos, BDNF, and pERK levels in the mPFC, amygdala, and hippocampus are shown in Fig. 5. The c-Fos levels were not altered by ketamine in any of those regions as shown in Fig. 5A (p > 0.05). The ketamine (10 mg/kg) infusion significantly increased BDNF levels in the amygdala (t = 2.37, p = 0.033) compared to the saline controls, while BDNF levels were not different in the mPFC and hippocampus (p > 0.05). The ketamine infusion reduced pERK levels in the mPFC (t = 2.81, p = 0.014) and hippocampus (t = 2.22, p = 0.044).

## 4 Discussion

Although there have been previous studies of ketamine in rodents, most studies used an IP ketamine injection which may limit the translational value to clinical practice. To our knowledge, there is no information available on the effects of IV ketamine infusions on the neural plasticity-related protein levels in rodent brains. Therefore, we administered low and high doses of IV ketamine (10 and 40 mg/kg) infusion over 2 h and measured protein levels 2 h after the infusion. The low dose ketamine (10 mg/kg) did not alter c-Fos levels, but increased BDNF levels in the amygdala and reduced pERK levels in the mPFC and hippocampus. On the contrary, the high dose ketamine (40 mg/kg) increased c-Fos levels in the mPFC and amygdala, and increased pERK levels in the mPFC and hippocampus. The fear conditioning alone did not alter any of these proteins levels when measured 4 h after the fear conditioning. The current findings suggest that sub-anesthetic IV ketamine infusions may produce dose-dependent and brain region-specific changes in protein levels that are implicated in neural plasticity and learning.

The 40 mg/kg ketamine infusion administered in the current study increased c-Fos levels in the mPFC and amygdala, suggesting that these regions are important for ketamine effects. Previous studies have demonstrated that NMDA glutamate receptor antagonists can increase c-Fos levels in multiple brain regions such as the amygdala, prefrontal cortex and hippocampus (Keilhoff et al., 2004; Pietersen et al., 2006; Peng et al., 2011). Since the mPFC and amygdala may work together to regulate fear behaviors (Barbas, 2000; Quirk et al., 2003; Ghashghaei et al., 2007), increased c-Fos levels in these regions suggest a high dose ketamine infusion may activate this regulatory network.

The mPFC may be a critical site for ketamine because the blockade of NMDA receptors in the mPFC leads to a range of behavioral and memory alterations (Nowak et al., 2012). In our previous study, IV ketamine infusions dose-dependently increased dissociative stereotypy behaviors in rats, which are thought to be regulated by the mPFC (Radford et al., 2017). Another study found that an intra-PFC injection of NMDA receptor antagonists produced working memory deficits in rodents (Nowak et al., 2012). In addition, ketamine-induced increases in c-Fos levels in the amygdala may indicate a propensity for enhanced fear learning in animals (Arnold et al., 1992; Pietersen et al., 2006). We recently observed increased fear memory retrieval and delayed fear extinction in rats that received an IV ketamine infusion after fear conditioning along with increased brain glucose uptake in the mPFC and amygdala (Radford et al., 2018a). Taken together, increased c-Fos levels in the mPFC and amygdala suggest that a high dose IV ketamine infusion impacts key regulatory brain regions for the rodent fear response.

The low dose ketamine infusion increased BDNF levels in the amygdala, while the high dose had no effect in the three regions sampled. We recently reported that IV ketamine infusions dose-dependently elevated CORT levels, while only the high dose of ketamine (40 mg/kg) decreased BDNF levels in the plasma samples of rats (Radford et al., 2018b). This indicates a discrepancy between plasma and brain BDNF levels following ketamine infusions. Previous studies reported increased BDNF levels in the brain after various doses of ketamine injection, and suggested this increase may be a marker for antidepressant effects of ketamine (Garcia et al., 2008b; Reus et al., 2011; Wang et al., 2011; Akinfiresoye and Tizabi, 2013; Yang et al., 2013; Zhou et al., 2014). However, other studies reported no changes in BDNF levels in the frontal cortex and hippocampus of rats after a single injection of ketamine (10 and 20 mg/kg, IP) (Goulart et al., 2010; Saur et al., 2017; Le Nedelec et al., 2018). Our results of no changes in BDNF levels in the mPFC and hippocampus after IV ketamine (10 and 40 mg/kg) are consistent with these findings. BDNF levels are known to undergo time-dependent changes following inescapable stress and ketamine administration (Bland et al., 2005; Choi et al., 2017). Therefore, various factors such as ketamine dosages, route of administration, and the timing of the BDNF assay after ketamine administration may contribute to the variable effects of ketamine on BDNF levels in the brain.

We found that low and high doses of IV ketamine infusion induced opposite effects on pERK levels in the mPFC and hippocampus when measured 2 h after the infusion. The extracellular signal-regulated kinase (ERK) has multiple roles in neurobiology to include learning and memory. The components of ERK1/2 have been thoroughly investigated and are believed to be involved in the mechanisms of synaptic plasticity (Sun and Nan, 2017). ERK activation is required for full expression of long-term potentiation (LTP) and is necessary for the consolidation of fear memory (Schafe et al., 2000; Duvarci et al., 2005; Villarreal and Barea-Rodriguez, 2006). Phosphorylation of ERK1/2 mediates signaling cascades, which influence the synthesis of a large number of proteins that change the structure and function of neurons (Koike et al., 2011). Previous studies have demonstrated that ketamine injection (10 mg/kg, IP) increased the phosphorylation of ERK in the mPFC (Li et al., 2010; Liu et al., 2013; Girgenti et al., 2017). However, another study reported no changes in pERK levels with ketamine (5 and 15 mg/kg, IP) administration in the cortex of mice (Salort et al., 2019). Interestingly, an IV ketamine (0.5 mg/kg) delivered over 4 s reduced pERK levels in the hippocampus of rats (Caffino et al., 2016). Moreover, morphine-induced increased pERK levels were blocked by intrathecal ketamine (1 μg) in the spinal cord of mice (Shen et al., 2018), suggesting that a low dose ketamine has the potential to reduce phosphorylation of ERK. In our study, the 10 mg/kg ketamine infusion reduced pERK levels while the 40 mg/kg ketamine increased it in the mPFC and dorsal hippocampus. Thus, low and high doses of IV ketamine infusion can produce opposite effects on pERK levels in brain regions that are critical for learning and memory.

Interestingly, mild fear conditioning alone did not alter any of the protein levels assayed in this study. Although c-Fos can be sensitive to stressful stimuli, studies also have shown that c-Fos expression is transient, reaching a peak expression between 60 and 120 min after the stimulus and gradually decreasing to the baseline levels after 4 h (Tulogdi et al., 2012). In the current study, we measured c-Fos levels 4 h after fear conditioning (2-h ketamine infusion and 2-h delay; Fig. 1A), therefore, we may have missed the timing of elevated c-Fos levels in the brain. Moreover, there was no significant interaction between fear conditioning and a high dose ketamine infusion, indicating minimal effects of fear conditioning on these protein levels. Overall, the primary purpose of the current study was to investigate the effects of IV ketamine infusion, rather than fear conditioning, on these protein levels in brain regions implicated in plasticity.

The current study is not without limitations. Given the time-dependent effects of ketamine on BDNF levels, it would be important to measure BDNF levels at multiple points following IV ketamine infusion, such as 24 h post-infusion similar to previous IP ketamine studies (Li et al., 2010; Burgdorf et al., 2013). We only studied male rats so that we cannot generalize the current findings to female rats. A future study is necessary to investigate molecular and functional significance of changes on these proteins following IV ketamine infusion.

## 5 Conclusions

We utilized the clinically relevant IV route of ketamine administration to measure effects on c-Fos, BDNF, and pERK protein levels in three brain regions that are critical for neural plasticity. The IV ketamine infusion induced dose-dependent and region-specific effects on those protein levels. Thus, it is crucial to test a wide range of ketamine doses in preclinical studies. A better understanding of the molecular mechanisms of IV ketamine infusions on different brain regions will enable us to contribute to the improved clinical practice and research regarding novel applications for ketamine treatment.

## Conflicts of interest

None. The views expressed in this article are those of the authors and do not reflect official policy or position of the Uniformed Services University of the Health Sciences, Department of the Navy, the Department of Defense, or the United States Government.
